# Soil propagule bank of ectomycorrhizal fungi associated with Masson pine (*Pinus massoniana*) grown in a manganese mine wasteland

**DOI:** 10.1371/journal.pone.0198628

**Published:** 2018-06-05

**Authors:** Jian Huang, Qisheng Han, Junjian Li

**Affiliations:** 1 College of Forestry, Northwest A&F University, Yangling, China; 2 Farmland Irrigation Research Institute, Chinese Academy of Agricultural Sciences, Xinxiang, China; 3 Institute of Loess Plateau, Shanxi University, Taiyuan, China; University of Roehampton, UNITED KINGDOM

## Abstract

Ectomycorrhizal (ECM) fungal propagule bank could facilitate the regeneration and plantation of seedlings in disturbed area. In this study, Masson pine (*Pinus massoniana*) seedlings were used to bait the ECM fungal propagule bank buried in the soils collected from a manganese (Mn) mine wasteland and a non-polluted area in China. After 6-month growth, we found the seedlings grown in the Mn mine soil (Mn:3200 mg kg^-1^) did not display any toxicity symptoms. Based on morphotyping and ITS-PCR sequencing, we identified a total of 16 ECM fungal OTUs (operative taxonomic units) at 97% similarity threshold, among which 11 OTUs were recovered in the Mn mine soils and 14 in the non-polluted soil. Two soil types shared 9 OTUs and both of them were dominated by a *Tylospora* sp. Based on those soil propagule banks in Masson pine forests reported in previous, we speculated that some Atheliaceae species may be preferred in the soil propagule bank of some pine species, such as Masson pine. In addition, NMDS ordination displayed geographical position effects on soil propagule banks in five Masson pine forest from three sites at regional scale. In conclusion, Masson pine ECM seedlings could grow well in the Mn wasteland as a suitable tree species used for reforestation application in Mn mineland, in addition, Mn pollution did not alter the dominant ECM fungal species in the soil propagule banks.

## Introduction

Heavy metal mining industry produces millions of tonnes of polluted mine tailings, and destructed ground vegetation severely. Ecological restoration of mine wastelands has attracted much attention all over the world. Forest reclamation with native tree species in the mine wastelands might be one of the ideal approach, which shows the advantages such as soil stability, watershed protection, and ecological benefits. Soil microbial communities are very important for successful reclamation of mined sites, such as the ectomycorrhizal (ECM) fungi [1, 2]. ECM symbiosis is developed between a special group soil fungi and woody plants, which is indispensable for the ECM tree growth in harsh conditions by improving the uptake of water and nutrients [3]. In addition, ECM symbiosis has been observed to help the host plant to detoxify heavy metals [4, 5], and regulate the expression level of genes involved in metal uptake and transport [6, 7]. To facilitate the process of reforestation, more knowledge about the ecological progress of ECM fungal communities in heavy-metal polluted mineland is desired.

In mineland, most of ECM roots and mycelia were diminished with the removing of forest coverage, and therefore resistant soil ECM fungal propagules would provide the inoculum sources for establishment of ECM symbiosis on regenerating seedlings and plantation [8, 9]. In the last decade, soil ECM fungal propagule bank in different forest types and sites have received much interest. ECM fungal spore banks were found to be a small subset of ECM fungal community in the mature forest, and showed homogenous in species composition [10]. In mineland, high level of heavy metals has adverse effects on ECM fungal community structure as well as the host trees [11, 12]. The existing information about the ECM fungal community in heavy-metal polluted soils is mostly based on the ECM root systems in field [13–16]. However, the structure of soil ECM fungal propagules remain poorly understood in the heavy-metal polluted sites.

Manganese is an essential nutrient element necessary for activation of a wide range of enzymes. High concentrations of Mn could interfere with calcium (Ca), magnesium (Mg), iron (Fe), phosphorus (P), etc. and disturb their absorption, translocation [17], excess Mn causes chlorosis and necrosis, the appearance of brown, necrotic spots or small reddish purple spots and sometimes, dark root tips [18]. ECM symbiosis could protect its host plants against the toxic effect of Manganese (Mn) [19]. Mn pollution could also affect fungal populations by reducing the abundance of some groups of ECM fungi [14, 16]. Despite the fact that Mn have low toxicity, some ECM fungi showed different tolerance to Mn stress in *vitro* [19, 20]. In our previous studies, we found that ECM fungal richness of soil propagule banks in Pb-Zn and Cu mine tailings were generally lower than those of the non-disturbed forests [16]. In addition, we observed some dominant species such as *Inocybe curvipes*, *Suillus luteus* associated with the Masson pine (*Pinus massoniana*) grown in Pb-Zn tailing and Cu mine tailing, respectively. In this study, we performed a seedling bioassay experiment to bait the ECM fungal propagules in a Mn mineland. We attempted to determine: (1) whether young seedlings of Masson pine could grow well in the Mn mine tailing soils, (2) whether the ECM fungal propagule bank was altered by Mn mine wastes, and are there some special ECM fungal species preferring to the Mn mine soil, (3) finally, we estimate ECM fungal species richness and geographic pattern of soil propagule banks of Masson pine forest from five Masson pine forest site.

## Materials and methods

### Site description

**Fig 1:**
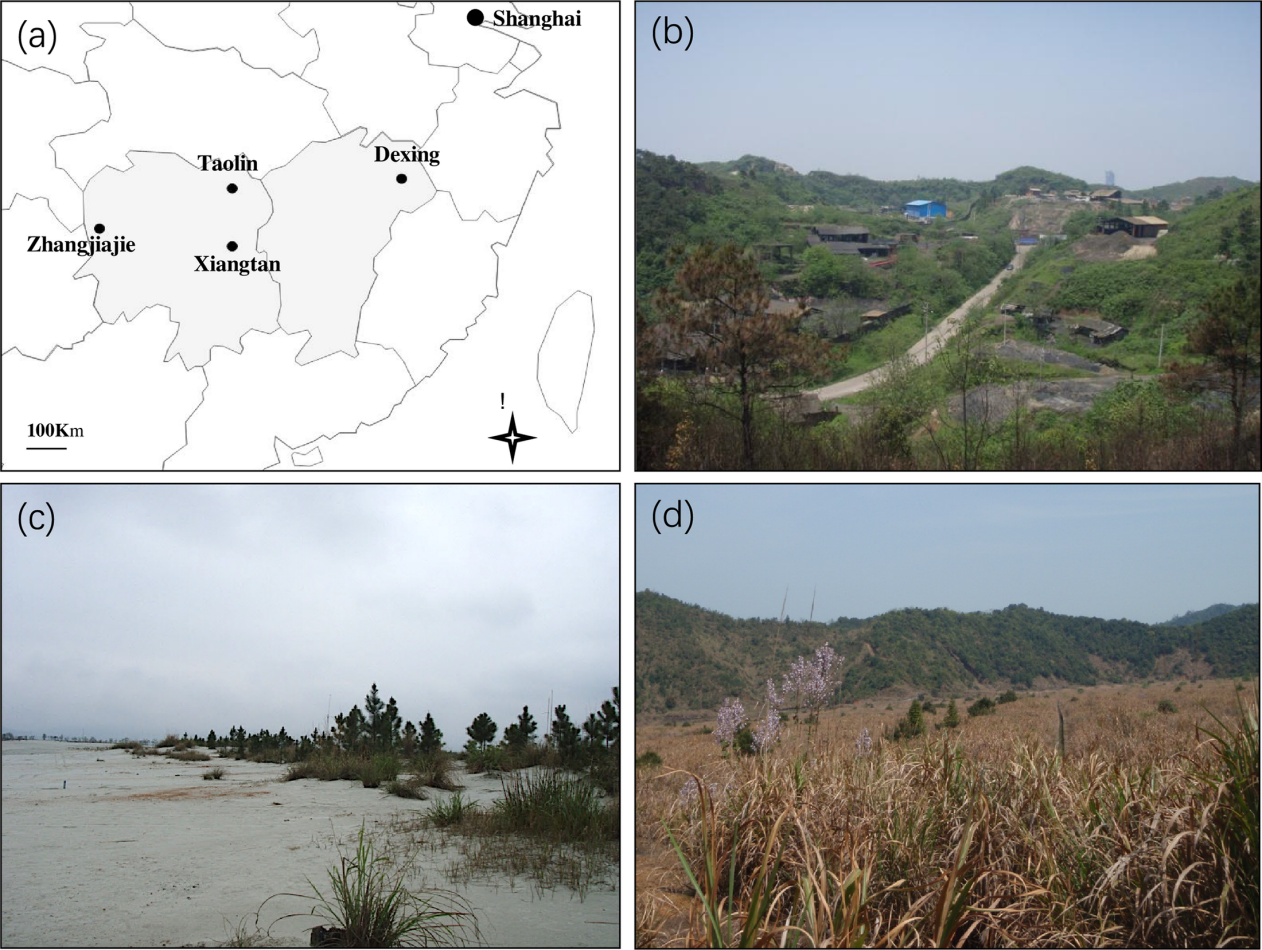
**a**, Position of study sites in China. **b**, Mn mineland in Xiangtan city. **c**, Pb-Zn tailing in Taoling. **d**, Cu tailing in Dexing city.

Mn contaminated soil sampling was performed in a Mn mine tailing area, which belonged to Xiangtan Mn mine (27.48° N, 112.85° E), Hunan Province, China. A natural secondary Masson pine forest stand (29.37° N, 113.63° E) in Linxiang City, Hunan Province, China, was selected as the reference forest. The positions of those sites were indicated in Fig 1a. Both two areas have been described in our previous studies [13, 14, 16]. Masson pine was one of dominant tree species in the regional forest stand at Hunan Province. In the Mn tailing area, some Masson pine (*Pinus massoniana*) and white oak (*Quercus fabri*) mix forest patches were remaining at the sampling time.

These sampling sites were located in the state-owned forests and no specific permissions were required for scientific research in these locations. In addition, our field studies did not involve endangered or protected species.

### Soil sampling

Sampling was performed in April, 2015. Soil samples were took from 30 randomly distributed spots with over five meters distance from each other in ca. 3 km×3 km core area of the Xiangtan Mn mine [14]. We took black soil samples in the Masson pine forest patches where Mn tailings were dumped on the forest floor. At each spot, ca.150 ml soil from three 2 m × 2 m (length × width) areas were collected, respectively using a trowel after removing all visible plant material and waste rocks on the forest floor and then mixed, stored in plastic zip-lock bags. On the other hand, the soil samples were collected from the non-polluted forest as described above. In the laboratory, the soil samples were firstly air-dried for one month and then subjected to sieve for removing the large particles and visible roots before seedling bioassay. Thirty pots were prepared for the Mn mine tailing soil and the reference (non-polluted) soil, respectively, and ca. 350 ml soil sample were put into one pot (diameter:15 cm; height: 15 cm). In addition, ~ 50 grams of soil from each pot was stored for chemical analysis. Seeds of Masson pine were surface sterilized (70% ethanol(v/v) for 1min, 6% calcium hypochloride for 30 min) prior to sow seeds. Two 45d-old seedlings were transplanted into each pot. One well-grown seedling was remained in each soil sample after first 45d of growth and then all seedlings were cultivated in a greenhouse for five months in total. To assess the airborne contamination, the seedlings were also planted in 10 pots filled with sterilized soil substrates and placed near to those bioassay seedlings.

### Molecular identification of ECM

Seedling roots were gently washed in tap water and observed for general morphological classification under a stereomicroscope (PXS9-T, CeWei photoelectric technology Co. Limited). The roots of the reference seedlings were also checked. The number of the root tips and ECM tips were counted for seedlings. The representative ECM tips (5–10 for both soils) for each ECM morphotype in one seedling were collected and frozen-dried for DNA extraction following the method described in Long *et al*. (2016)[21]. Briefly, each individual ECM root tip was put into a 2-ml centrifuge tube with a stainless-steel ball for pulverizing (MM400, Restch, Germany). The cetyltrimethyl ammonium bromide (CTAB) method was used to extract DNA. Polymerase chain reaction (PCR) was conducted to amplify the internal transcribed spacer (ITS) region of rDNA of ECM fungi using the Taq MasterMix (Kangwei, Beijing, China), and using the primer set of ITS1F and ITS4 [22]. The amplified PCR products were checked on 1.5% agarose gels and visualized under UV light to examine the quality and quantity of amplified bands with Bio-Rad Gel Doc^™^ XR+ system (Bio-Rad, CA, USA). Sequencing was conducted on ABI Prism 3730xl genetic analyzer (Applied Biosystems, Foster City, CA) using ITS1F / ITS4. High-quality sequences were aligned and delimited into molecular operational taxonomic units (OTUs) at 97% sequence similarity threshold. The sequences were compared with a known sequence for species delimitation in the UNITE database / NCBI database. All unique sequences were deposited to DNA Data Bank of Japan (DDBJ) under the accession number LC176630-LC176645.

### Soil and plant chemical analysis

After measuring the biomass and length, seedlings were dried at 80 °C for 48 h and cut into two parts: shoot (stem and needles) and root. The plant samples were digested in a high-purity acid mixture HNO_3_:HClO_4_ (5:1) at 150°C until digested completely. Concentrations of manganese (Mn), copper (Cu), lead (Pb) and nickel (Ni) in the solutions were determined using an atomic absorbance instrument (AA-7000, Beijing East and West Analysis Instrument Co., Ltd). The metal concentrations of the soils were measured as described above.

### Statistical analysis

The Student’s t-test was used to test the significant differences of heavy metal content between the soils and plant parts (shoot and root). The ECM colonization rate was calculated (number of ECM root tips / total number of the root tips in each seedling). The ECM fungal richness per seedling was the number of ECM fungal OTUs detected in the root of a single seedling. The relative abundance of each OTU was calculated (number of tips of a certain OTU / total number of ECM tips in each soil type). The frequency of each ECM fungal OTU was calculated as the ratio of number of seedlings colonized by the each OTU to 30 pots. Chao2 richness estimations of ECM fungi were calculated for the both Mn mine soil and non-polluted soil using Estimate S [23]. ECM fungal diversity in the Mn mine soil and non-polluted soils were compared with Shannon, Simpson and Pielou indices. To evaluate the sampling efforts, we built the accumulation curves for the samples in present study and three other propagule banks from a Taoling Pb-Zn tailing in Linxiang City, a Cu tailing and secondary forest in Dexing city reported in previous [16]. Nonmetric multidimensional scaling analysis (NMDS) was performed to visualize the dissimilarity of the five ECM fungal propagule bank as mentioned in above using the package ‘vegan’ in R, and then the function of ‘envfit’ was used to test the effects of site and soil factors on the community structure. Mantel test was used to evaluate whether the geographical distances were correlated with the Bray–Curtis distances of ECM fungal composition among the five sites. All statistical analyses were carried out in R 3.2.2 [24].

## Results

### Seedling growth

The soil samples from the Mn mine tailings had higher concentrations of Mn, Pb, Cu and Ni than the soil samples from the reference site (Table 1). In particular, soils from the Mn mine sites contained high concentrations of Mn (mean 3200 mg kg^-1^), which was nearly ten times higher than that in the reference (Table 1).

**Table 1:** Heavy metal concentration of soil samples from Mn-mine tailing and non-polluted reference.

Soil type	Mn (mg kg^-1^)	Pb (mg kg^-1^)	Cu (mg kg^-1^)	Zn (mg kg^-1^)	Ni (mg kg^-1^)
Mn mine tailing	3224.80 ± 293.9*	213.90 ± 22.92*	44.08 ± 1.78*	77.71 ± 25.86	84.97 ± 11.44
Non polluted soil	334.95 ± 170.62	110.31 ± 54.06	23.59 ± 10	194.48 ± 65.93*	50.91 ± 40.86

The values are Means ± standard errors;

* significant difference (*P* < 0.05);

After five-months growing in the black Mn mine soil, the seedlings did not show any obvious toxicity symptoms, such as chlorosis, retarded growth, and red needles. The biomass of the seedlings grown in the Mn mine soil (1.62 ± 0.85 g, mean ± SE, *n* = 30) was not significantly different from those grown in the non-polluted soil (1.65 ± 0.61 g, mean ± SE, *n* = 30) (Table 2). In addition, no significant difference in the shoot length was observed between the Mn mine soil (12.10 ± 3.00 cm) and the non-polluted soil (11.70 ± 2.50 cm).

**Table 2:** Heavy metal concentrations of *Pinus massoniana* seedling grown in the Mn-mine tailing and non-polluted soils.

Plant parts	Soil type	Mn (mg kg-1)	Pb (mg kg-1)	Cu (mg kg-1)	Zn (mg kg-1)	Ni (mg kg-1)
root	Mn mine tailing	871.83 ± 864.58*	292.50 ± 282.79	21.15 ± 12.74*	71.86 ± 60.53	110.58 ± 109.52*
shoot	Mn mine tailing	131.57 ± 118.66*	141.22 ± 104.59	7.34 ± 4.62	30.57 ± 19.62	26.30 ± 25.37*
root	Non-polluted soil	98.93 ± 69.93	127.24 ± 120.85	17.26 ± 11.05	140.66 ± 95.28	69.04 ± 66.29
shoot	Non-polluted soil	28.31 ± 28.22	77.05 ± 75.03	6.43 ± 6.25	85.75 ± 46.07	1.16 ± 0.13

The values are Means ± standard errors;

* significant difference (*P* < 0.05)

Both shoot and root parts of seedlings grown in the Mn mine soils generally accumulated more heavy metals than those grown in the non-polluted soils (Table 2). In addition, the roots of seedlings accumulated more heavy metals than the shoots both in the Mn mine and non-polluted soils. In the Mn mine soil, particularly, the concentration of Mn in the roots (902 ± 375 mg kg^-1^) was about seven times higher than the shoots (131 ± 118 mg kg^-1^). In addition, seedlings grown in the polluted soils accumulated more Cu, Ni in roots and Ni in shoots than those grown in the non-polluted soils.

### Ectomycorrhizal identification

After five months of seedling growth, ECM had developed in the seedling roots. We counted on average 75–80 fine root tips per seedling. The seedlings grown in the Mn mine soils showed significantly lower ECM colonization rate (53.92 ± 12.32%) than those grown in the non-polluted soil (61.44 ± 13.72%) (*P* < 0.01).

**Fig 2:**
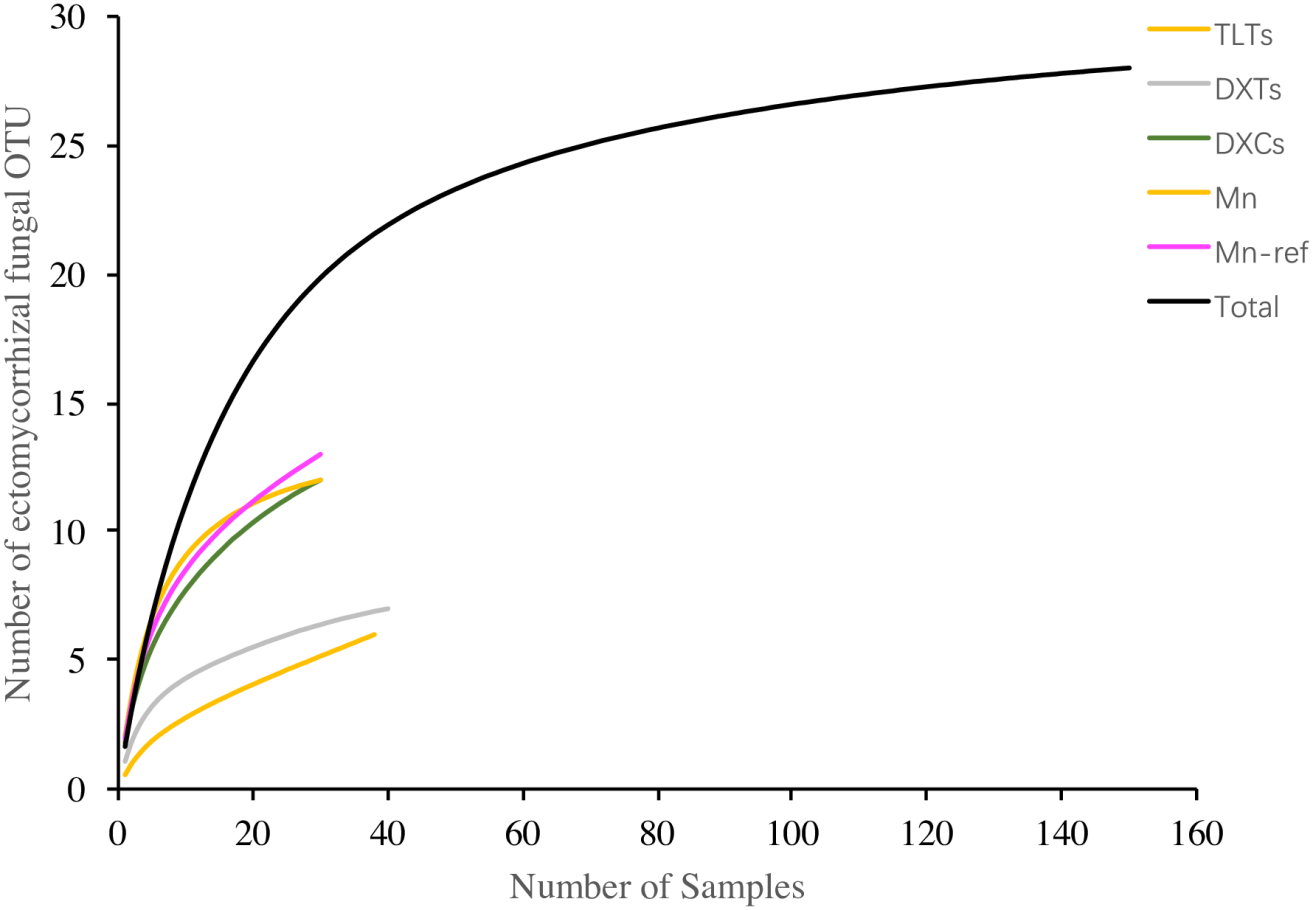
Accumulation curves for the richness of ECM fungal propagules baited by *Pinus massoniana* seedlings in the forest soils from a Mn mine wasteland in Xiangtan (Mn) and non-disturbed Masson pine forest in Linxiang city (Mn-ref), Pb-Zn tailing in Linxiang city (TLTs), Cu tailing in Dexing city (DXTs) and a non- disturbed Masson pine forest in Dexing city (DXCs) of China.

After ITS-PCR and sequencing analyses, a total of 16 OTUs of ECM fungi at 97% similarity threshold associated with the seedlings roots were identified (Table 3). These ECM fungal OTUs belonged to 10 genera and 9 families, and Basidiomycota dominated (11 OTUs) over Ascomycota (5 OTUs). Major taxa recorded in the study included: Thelephoraceae (5 OTUs), *Cenococcum* (3 OTUs) and Atheliaceae (2 OTUs). The seedlings grown in the Mn mine soils had 11 OTUs, while the reference had 14 OTUs. The estimated richness (Chao 2) in the Mn mine soil was also lower than in the non-polluted soil. The richness per seedling was 1.39 ± 0.55 in the Mn mine soils and 1.47 ± 0.56 in non-polluted soils, the difference was not statistically significant. In addition, no significant differences were observed between two soil types in the ECM fungal diversity indices (Table 4). The rarefaction curves of two communities in present study and the secondary forest in Dexing displayed similar pattern and showed greater increasing potential than those of the two spore banks in two tailings (Pb-Zn tailing and Cu tailing). After combining all bioassay seedlings (150 seedlings) together, the rarefaction curve showed more robust than each site and but nearly reached the plateau at the end (Fig 2).

**Table 3:** Identification of ectomycorrhizal fungal operational taxonomic units (OTUs) from the propagule bioassay *Pinus massoniana* seedlings.

OTUs	Accession number	Blast match voucher ID	Similarity	Relative abundance (%) / Frequency (%)	
				Mn tailing	Non-polluted soil
*Cenococcum* sp.1	LC176630	AB769888	466/467 (99%)	4.4 / 10.61	0.77 / 2.94
*Cenococcum* sp.2	LC176631	AB769889	970/970(100%)	8.65 / 21.21	0.12 / 1.47
*Cenococcum* sp.3	LC176632	LC095082	929/990(94%)	0 / 0	0.69 / 1.47
*Lactarius kesiyae*	LC176633	KR025621	649/650(99%)	0 / 0	12.39 / 20.59
*Neurospora* sp.	LC176634	FJ176470	536/538(99%)	0 / 0	0.12 / 1.47
*Pisolithus* sp.	LC176635	AB106874	614/624(98%)	1.77 / 1.52	5.47 / 7.35
*Pulveroboletus* sp.	LC176636	JQ991773	663/668(99%)	0 / 0	2.03 / 4.41
*Rhizopogon* sp.	LC176637	LC096919	704/705(99%)	1.35 / 3.03	15.88 / 27.94
*Thelephora terrestris*	LC176638	AB634267	600/601(99%)	4.7 / 6.06	0 / 0
*Tomentella* sp.1	LC176639	EF619823	562/574(98%)	10.94 / 12.12	5.75 / 5.88
*Tomentella* sp.2	LC176640	KC686883	601/605(99%)	18.65 / 19.7	13.16 / 16.18
*Tomentella* sp.3	LC176641	KP866136	600/603(99%)	2.9 / 3.03	4.05 / 5.88
*Tomentella* sp.4	LC176642	AB972839	540/541(99%)	0 / 0	8.59 / 14.71
*Tuber* sp.	LC176643	AB769932	512/512(100%)	18.73 / 21.21	0.2 / 1.47
*Tylospora* sp.1	LC176644	KF007260	472/505(93%)	21.47 / 30.3	30.78 / 25.29
*Tylospora* sp.2	LC176645	AB456674	545/547(99%)	6.43 / 10.61	0 / 0

**Table 4:** ECM fungal diversity indices for the Mn mine and non-polluted soil.

Study sites	Shannon index	Simpson index	Pielou index
Mn mine tailing	2.1	6.89	0.87
Non-polluted soil	2.02	5.94	0.76

**Fig 3:**
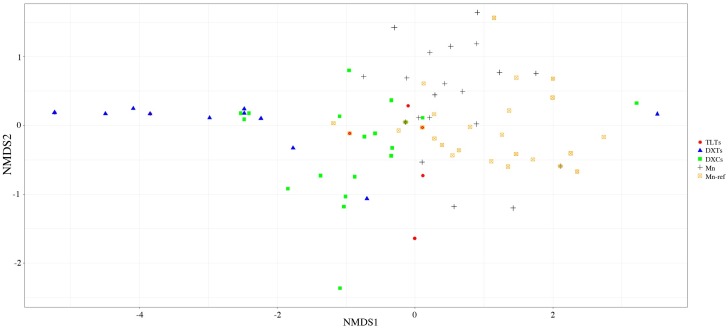
Non-metric multidimensional scaling ordination of soil propagule banks of ectomycorrhizal fungi in Mn mine soil and non-polluted soil and other three propagule banks (TLTs, DXTs, DXCs).

Among the 16 ECM fungal OTUs, 9 OTUs were common in two soil types (Table 3). Two OTUs (*Tylospora* sp.2 and *Thelephora terrestris*) were found only in the Mn soil and five OTUs (*Cenococcum* sp.3, *Lactarius kesiyae*, *Neurospora* sp., *Pulveroboletus* sp. and *Tomentella* sp.4) were found in non-polluted soil. *Tylospora* sp. 1 was the most dominant OTU in both two soil types. *Tuber* sp. (18.73%, relative abundance), *Tomentella* sp.1 (10.94%) and *Cenococcum* sp.2 (8.65%) were the dominant species in the Mn mine soil. In contrast, *Rhizopogon* sp. (15.88%, relative abundance), *L*. *kesiyae* (12.39%) and *Tomentella* sp.4 (8.59%) were the dominant species in the non-polluted soils (Table 2). Mantel test did not detect a significant correlation between the geographical distance and the similarity among five propagule banks. But, we can found the samples from Dexing were separated from those of other three sites. NMDS ordination of the present two banks and three other spore banks (Dexing Cu tailing, Dexing nature forest and Taolin Pb-Zn tailing) displayed that ECM fungal propagule banks associated with Masson pine forest showed geographical distribution pattern which was supported by the envfit test(*r* = 0.014), but not structured by the soil factors (Fig 3).

By comparison of the ECM fungal composition between the soil propagule bank and the corresponding field community (Huang et al., 2014), we found the OTUs richness were sharply decreased from the 43 OTUs in field to 16 OTUs in the propagule bank. We found 13 of 16 OTUs excepting three OTUs (*Neurospora* sp., *Pulveroboletus* sp., *Tuber* sp.) have the same lineage in the field. Two OTUs (*Rhizopogon* sp., and *Tylospora* sp.) were baited in the soil propagule bank, but absent from the field communities. Most of those OTUs in the propagule bank were also presented in the field communities with top rank in relative abundance. In particular, Atheliaceae sp., *Cenococcum* sp., and *T*. *terrestris* were abundant in both communities (S1 Table).

## Discussion

In this study, we used seedling bioassay to investigate the ECM fungal propagule banks in a Mn mine soil and non-polluted soil. The seedlings grown in the Mn mine wastes containing high content of Mn (mean 3200 mg kg^-1^). Phytotoxicities have generally been associated with 3–8 mg/kg total Cd in soil, 60–125 mg/kg Cu, 1500–3000 mg/kg Mn [25]. However, the young pine seedlings grown in the Mn tailings did not show any toxicity symptoms in this experiment. In contrast, seedlings grown in the Cu tailing and Pb-Zn tailings showed toxic symptoms [16]. It suggested that Masson pine seedlings were well adapted to in Mn-contaminated mine wastes. On the other hand, Mn is an essential trace element for plant growth and low toxicity to the plants even at high level [26].

ECM fungal spore banks in pine forests were often dominated by a limited set of fungal genera, geographically patterned at regional scales [10]. In this study, the ECM fungal composition was similar to our previous report in the Masson pine forests. We found Atheliaceae, *Rhizopogon*, *Cenococcum*, and *Tomentella-Thelephora* were major components of those soil propagule banks in Masson pine forests. Atheliaceae species were abundant in both propagule banks and the filed communities in Masson pine forest. It seems that Atheliaceae sp. have advantage in seedling colonization because they often form extensive mycelial systems [27]. *Tylospora* sp. was rare in propagule banks baited with several tree species, such as *P*. *densiflora* and *Betula maximowicziana* [28], *P*. *muricata* in fire-disturbed forest soils [28, 29], *Pseudotsuga sinensis* [30] and *P*. *bungeana* [31], but abundant in some pine forests at north America [10]. Thus, we think that Atheliaceae species might prefer to the soil propagule bank of some pine forest, such as Masson pine. In contrast, *Cenococcum*, *Rhizopogon* and *Thelephora-Tomentella* were frequently found in spore banks from different geographical distribution and forest type, which indicated these taxa are general taxa in the soil propagule banks. Miyamoto and Nara (2016) have revealed a strong effect of host tree species in determining the propagule banks of ECM fungi by investigating the soil spore banks from the different elevation on Mont. Ishizuchi [28]. However, Glassman et al., (2015) drawn a conclusion that ECM fungal spore banks correlated strongly with biogeographic location, but not with the identity of congeneric plant hosts at continental scale [10]. In this study, we also found a geographic pattern among the five propagule banks in Masson pine forests, the propagule banks from Dexing city were almost separated from other sites (Fig 3). Dexing are ~ 500 Km far from Taolin and Xiangtan while the distance between Taolin and Xiangtan is only ~170 Km. Taken together, we proposed that soil spore bank of ECM fungi would be divergent with the increasing of geographical distance or phylogenetic distance of host species.

We found the ECM fungal propagule bank in the Mn mine soil had the same dominant species (*Tylospora* sp. 1) as the non-polluted soil. In contrast, ECM fungal propagule banks in the Pb-Zn and Cu mine tailings had their unique dominant species [16]. In addition, most of the abundant ECM fungi baited by bioassay seedlings in propagule bank were also abundant in the field ECM fungal community (S1 Table). We found *Rhizopogon*, a dominant genus in soil spore banks [10, 32], was abundant in the non-polluted soils while few in the Mn polluted soils. In contrast, one Russulaceae member, *L*. *kesiyae*, was abundant (12%) in non-polluted soil while absent in the soil ECM fungal propagule bank from Mn-polluted soils. In addition, Russulaceae were found to be decreased with increasing Mn concentration in the field [14]. It indicated that the propagules of Russulaceae may be short-lived or sensitive to Mn toxicity. Besides, we identified an endophytic fungus, *Neurospora* sp. in several seedlings grown in the non-polluted soils. Spores of *Neurospora* were often activated by heat, and yet can also be stimulated to germinate in a variety of other conditions that involve carbohydrate sources [33]. Furthermore, we found the seedlings grown in the Mn mine wastes showed lower mycorrhizal rates than those in non-polluted soils, which suggested that Mn mine waste dressing decreased density of soil propagules or have toxic effect on mycorrhizal colonization. According to these founds, we proposed that soil propagule bank was less influenced by Mn pollution than the corresponding ECM fungal community.

## Supporting information

S1 Table Ectomycorrhizal fungi identified on Pinus massoniana and Quercus fabri growing in the Xiangtan Mn mining area of Hunan Province, China. (XLSX)
